# Site-specific characterization of SARS-CoV-2 spike glycoprotein receptor-binding domain

**DOI:** 10.1093/glycob/cwaa085

**Published:** 2020-09-03

**Authors:** Aristotelis Antonopoulos, Steven Broome, Victor Sharov, Christopher Ziegenfuss, Richard L Easton, Maria Panico, Anne Dell, Howard R Morris, Stuart M Haslam

**Affiliations:** Department of Life Sciences, Imperial College London, London, SW7 2AZ, UK; BioPharmaSpec Inc, 363 Phoenixville Pike, Malvern, PA 19355, USA; BioPharmaSpec Inc, 363 Phoenixville Pike, Malvern, PA 19355, USA; BioPharmaSpec Inc, 363 Phoenixville Pike, Malvern, PA 19355, USA; BioPharmaSpec Ltd, Suite 3.1, Lido Medical Centre, St. Saviour, Jersey, JE2 7LA, UK; Department of Life Sciences, Imperial College London, London, SW7 2AZ, UK; BioPharmaSpec Inc, 363 Phoenixville Pike, Malvern, PA 19355, USA; BioPharmaSpec Ltd, Suite 3.1, Lido Medical Centre, St. Saviour, Jersey, JE2 7LA, UK; Department of Life Sciences, Imperial College London, London, SW7 2AZ, UK; Department of Life Sciences, Imperial College London, London, SW7 2AZ, UK; BioPharmaSpec Inc, 363 Phoenixville Pike, Malvern, PA 19355, USA; BioPharmaSpec Ltd, Suite 3.1, Lido Medical Centre, St. Saviour, Jersey, JE2 7LA, UK; Department of Life Sciences, Imperial College London, London, SW7 2AZ, UK

**Keywords:** glycoproteomics, mass spectrometry, SARS-CoV-2, spike glycoprotein

## Abstract

The novel coronavirus SARS-CoV-2, the infective agent causing COVID-19, is having a global impact both in terms of human disease as well as socially and economically. Its heavily glycosylated spike glycoprotein is fundamental for the infection process, via its receptor-binding domains interaction with the glycoprotein angiotensin-converting enzyme 2 on human cell surfaces. We therefore utilized an integrated glycomic and glycoproteomic analytical strategy to characterize both N- and O- glycan site-specific glycosylation within the receptor-binding domain. We demonstrate the presence of complex-type N-glycans with unusual fucosylated LacdiNAc at both sites N331 and N343 and a single site of O-glycosylation on T323.

## Introduction

The coronavirus disease 2019 (COVID-19) pandemic caused by the severe acute respiratory syndrome coronavirus 2 (SARS-CoV-2) virus is having a global impact with latest WHO data indicating 21,294,845 cases and 761,779 deaths (WHO COVID-19 Situation Report 209). The spike glycoprotein of SARS-CoV-2 plays a vital role in facilitating viral infectivity of host cells. It exists as a homotrimer on the viral surface. Each monomer comprises an S1 subunit, which contains the host cell receptor-binding domain (RBD), and a membrane-anchored S2 subunit involved in fusion of viral and host membranes which are formed by proteolytic processing (Walls et al. 2020). The RBD is responsible for binding angiotensin-converting enzyme 2 (ACE2) receptor on human cell surfaces (Shang et al. 2020). Informatic analysis of the spike glycoprotein derived amino acid sequence predicts it to be heavily N- and O-glycosylate. Such heavy glycosylation is likely to be functionally important for host cell recognition and viral entry, immune system interactions and any potential future vaccine or diagnostic developments (Kumar et al. 2020).

Rapid progress has already been made in characterizing spike glycoproteins glycosylation with two detailed glycoproteomic studies (Shajahan et al. 2020; Watanabe et al. 2020). However, there is variability between the presented structural data. For example, Shajahan et al. (2020) indicate that site N616 expresses only high mannose glycans, whereas Watanabe et al., indicate that this site expresses mostly complex N-glycans with only traces of high mannose and hybrid N-glycans. This clearly indicates both the complexity of glycoproteomic analysis, but also the potential impact that the structure of the spike glycoprotein recombinant constructs and bioprocessing conditions can have on recombinant glycoprotein glycosylation.

We used an integrated glycomic and glycoproteomic strategy to characterize the N- and O-glycosylation of recombinant SARS-CoV-2, Spike glycoprotein S1 Subunit, RBD (Arg319-Phe541) expressed in HEK293 cells. We demonstrate the presence of complex-type N-glycans with unusual fucosylated LacdiNAc and a single site of O-glycosylation on Thr323 at the beginning of the RBD. We discuss our data in the context of previous spike glycoprotein characterizations, but also highlight the limitations of the characterization of recombinant spike glycoproteins.

## Materials and methods

### Sample Information

Recombinant SARS-CoV-2, Spike protein S1 Subunit RBD (Arg319-Phe541) derived from transfected human HEK293 cells was obtained from RayBiotech (Georgia, Product Number 230-30162).

### Sample digestion

The sample was buffer exchanged into 0.1 M Tris/3 M guanidine HCl (pH 8.5) and incubated with 25 mM dithiothreitol (DTT) at 37 °C for 30 min. The reduced sample was carboxymethylated by addition of 0.1 M Iodoacetic acid and reacted at room temperature in the dark for 30 min. The reduced and carboxymethylated sample was buffer exchanged into 50 mM ammonium bicarbonate, pH 8.4.

A two-stage enzymatic digestion strategy was employed. Initially by digested with Trypsin (Roche p/n 11418475001) (1:50 w/w enzyme:protein ratio) at 37°C for 3 h, then after adjustment to pH 4 the sample was subjected to Glu-C digestion (Roche p/n 11047817001) (1:20 w/w enzyme:protein ratio) at 37°C for 3 h.

### Glycoproteomics analysis of digested samples

LC ES-MS (MS^e^) and collisionally activated CAD MS/MS (DDA) analyses were performed on the products of digestion using an Acquity UPLC coupled with a Xevo G2-XS Q-TOF Mass Spectrometer (Waters, Milford, MA). MS^e^ is a feature of the Waters Q-TOF mass spectrometer acquisition control allowing alternating generation of total fragment ion data from the ion beam, switching between high and low collision energies, without the prior individual mass selection of CAD MS/MS. Separations were achieved on the Acquity UPLC using a 100 mm × 2.1 mm C_18_ reversed phase column eluted with a linear gradient of 0.1% formic acid in water (Buffer A) and 0.1% formic acid in acetonitrile (Buffer B) at a flow rate of 0.4 mL/min. The gradient used was: 0 min, 0% B; 2 min, 0% B; 62 min, 26% B; 68 min, 31% B; 74 min, 45% B; 74.5 min, 85% B and 79.5 min, 85% B. Fractions corresponding to the three observed glycopeptide regions of the chromatogram, identified using carbohydrate reporter fragment ions at m/z 204, 274, 292 and 366 in an initial analytical run, were manually collected for further study by stream-splitting the LC eluent in a second LC–MS run (11–17 min for 341-VFNATR-346, 18–24 min for 320-VQPTESIVR-328, 48–65 min for 329-FPNITNLCPFGE-340). Interpretations of glycopeptide ESMS and ESMS/MS data were performed manually by previously reported methods based on amino acid and sugar masses together with the known fragmentation mechanisms of biopolymers (Dell and Morris 2001; Panico et al. 2016). Collected fractions for subsequent glycomic analysis or additional digestion were taken to dryness on a Speed-Vac (Thermo Fisher). The theoretical masses of identified peptides and glycopeptides were calculated using MassLynx or ProteinProspector V 6.2.1.

**Fig. 1:**
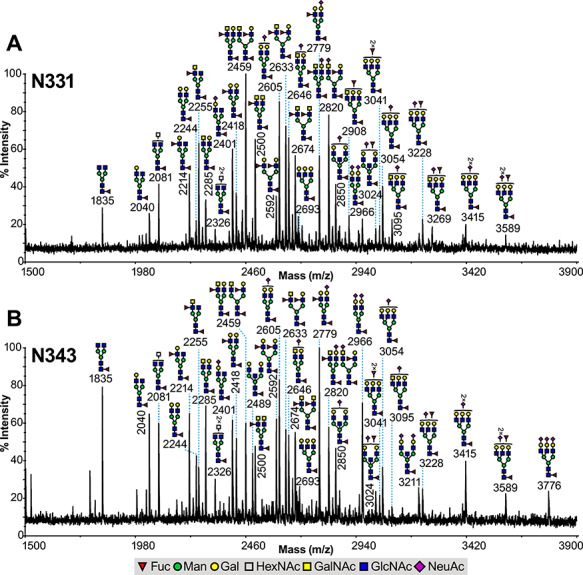
Glycomic analysis of the N331 and N343 glycopeptides derived from the RBD of the S1 spike SARS-CoV-2 glycoprotein. MALDI-TOF mass spectra of permethylated N-glycans derived from (**A**) 329-FPNITNLCPFGE-340 glycopeptide (containing the N331 glycosylation site) and (**B**) 341-VFNATR-346 glycopeptide (containing the N343 glycosylation site). All signals observed are singly charged sodiated ions [M + Na]^+^ and their structural assignment is based on monosaccharide composition, MS/MS fragmentation analyses and knowledge of the biosynthetic pathways. Residues shown on top of a bracket have not had their antenna location unequivocally defined.

### Preparation of N-glycans

The isolated fractions identified in the LC–MS analyses as containing N-linked glycopeptides were dissolved in 50 mM ammonium bicarbonate, pH 8.4, to which was added 1 U of PNGase F (Roche p/n 11365169001) and incubated at 37°C for 20 h. Released N-glycans were separated from the peptides using an RP C_18_ Sep-Pak and permethylated (Ciucanu and Kerek, 1984).

### Analysis of permethylated N-glycans

Permethylated samples were dissolved in 10 μL of 50% methanol in water, 1 μL was mixed with 1 μL of 2,5-dihydroxybenzoic acid (10 mg/mL in 50% methanol in water). MALDI data were acquired in the positive ion reflector mode using a 5800 mass spectrometer (AB Sciex, Framingham MA). The MALDI data were analyzed using Data Explorer 4.9 (AB Sciex). The processed spectra were subjected to manual assignment and annotation with the aid of GlycoWorkBench (Ceroni et al. 2008). The proposed assignments for the selected peaks were based on composition together with knowledge of the biosynthetic pathways. Proposed structures were further confirmed by MS/MS analysis.

## Results

### Glycomics and glycoproteomics analysis of the N331 and N343 glycosylation sites

The recombinant SARS-CoV-2 S1 RBD expressed in HEK293 cells was subjected to a two-stage enzymatic digestion strategy employing Trypsin, followed by a Glu-C digestion in order to allow separation of the two N-linked consensus site peptides which would otherwise be present within a single tryptic peptide. The resulting glycopeptides were purified to allow separation of the 329-FPNITNLCPFGE-340 and 341-VFNATR-346 glycopeptides which contain the glycosylation sites N331 and N343, respectively (hereafter referred as N331 and N343 glycopeptides). For the glycoproteomic analysis, on-line ES-LCMS and MS/MS of the digest samples was achieved in separate runs for both MS^e^ and CAD MS/MS, and initial interpretation showed the presence of complex core-fucosylated N-glycans at both sites, including fucosylated LacdiNAc antennal structures identified by their distinctive fragment ions (see later). In parallel work, the glycomic analysis of the purified N331 glycopeptide showed that the N331 glycosylation site contained exclusively complex N-glycan structures with compositions consistent with core-fucosylation and ranging from bi- to tri-antennary (Figure 1A). Interestingly, as with the independently interpreted LC–MS data, the antennae of these complex N-glycans were found to contain LacdiNAc (GalNAcβ1-4GlcNAc) units, either on all of their antennae, occurring on bi-antennary N-glycans (m/z 2500 and 2674), or mixed with *N*-acetyllactosamine (LacNAc) units, occurring on bi- (m/z 2285, 2459, 2633, 2646 and 2820) and tri-antennary (m/z 2908, 3095 and 3269) N-glycans. These complex N-glycans were found to be either undecorated (m/z 2081 and 2244 and 2285), fucosylated (m/z 2255, 2418, 2459 and 2500) and/or sialylated (m/z 2605, 2646, 2820, 3054 and 3095). Supporting evidence for the presence of N-glycans with LacdiNAc antennae was obtained from MALDI-TOF/TOF MS/MS analysis on selected molecular ions. For example, when the molecular ions at m/z 2459 and 2674 (Figure 1A) were subjected to MS/MS, they produced fragment ions at m/z 1780 and 1995, respectively, both corresponding to loss of fucosylated LacdiNAc from the molecular ion and both spectra contained the corresponding fragment ion at m/z 701 for fucosylated LacdiNAc (Supplementary Figure S1). In addition, some N-glycans were fully or partially agalactosylated (m/z 1835, 2040 and 2214) structures. Glycomics analysis on the purified N343 glycopeptide indicated that the N343 glycosylation exhibited similar types of N-glycan structures, with again exclusively complex structures, and minor differences in their relative abundance (Figure 1B). Again, of note is the presence of fucosylated LacdiNAc N-glycan structures.

**Fig. 2:**
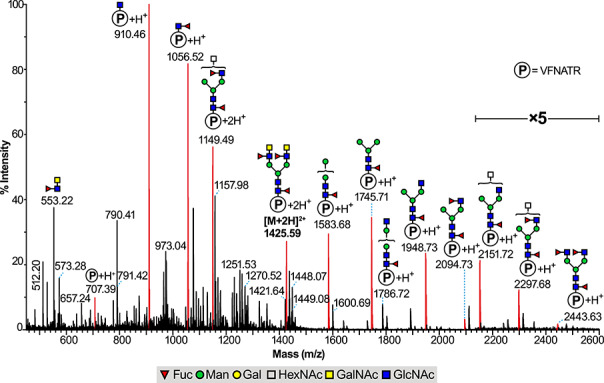
Glycoproteomics analysis of the 341-VFNATR-346 glycopeptide derived from the RBD of the S1 spike SARS-CoV-2 glycoprotein containing the glycosylation site N343 found centered at 12.1 min in the LC elution profile. LC ES-MS (MS^e^ mode) containing the [M + 2H]^2+^ molecular ion found at m/z 1425.59 and corresponding to the 341-VFNATR-346 glycopeptide with a core-fucosylated bi-antennary N-glycan with fucosylated LacdiNAc antennae (permethylated N-glycan found at m/z 2674 on Figure 1B and Supplementary Table SI). The peaks in red correspond to glycopeptide fragment ions deriving from the [M + 2H]^2+^ molecular ion. “P” inside a circle corresponds to the peptide backbone of 341-VFNATR-346. Area corresponding to m/z 2120 to 2600 has a 5-fold magnification. The residues shown on top of a bracket have not had their antenna location unequivocally defined.

The Glycoproteomic analysis of the N343 glycopeptide region of the chromatogram from the normal (low energy) and MS^e^ channels showed that it was glycosylated with various N-glycan compositions (Supplementary Table SI) which matched the structures obtained from the glycomics analysis (Figure 1B), including the LacdiNAc structures. This is exemplified by the LC-ES-MS (MS^e^), spectrum shown in Figure 2 where the major fragment ions can be interpreted as deriving from fragmentation of the glycopeptide doubly charged molecular ion [M + 2H]^2+^ detected at m/z 1425.59 corresponding to the bi-antennary core-fucosylated N-glycan with two fucosylated LacdiNAc antennae (Figure 2). The spectrum contains structurally informative fragment ions (amongst others) at m/z 910.46, 1056.52, 1583.68, 1745.71,1948.73, 2151.72, 2297.68 and 2443.63, and a characteristic reporter ion for the fucosylated LacdiNAc epitope at m/z 553.22 (Figure 2). Similar glycoproteomic analysis on the purified N331 glycopeptide exhibited the presence of various N-glycan compositions, which again matched the N-glycan compositions of the structures detected in the Glycomics analysis (Supplementary Table SII).

### O-linked glycosylation

**Fig. 3:**
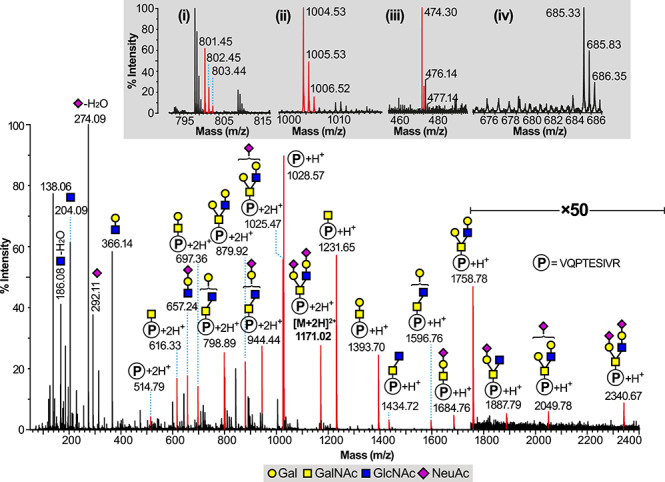
Glycoproteomics analysis of the 320-VQPTESIVR-328 glycopeptide containing the putative glycosylation site at T325 and S325 derived from the RBD of the S1 spike SARS-CoV-2 glycoprotein. LC ES-MS (MS^e^ mode) data containing the [M + 2H]^2+^ molecular ion found at m/z 1171.02 and interpreted as a single Core 2 substitution at Thr 323 with the HexNAc_2_Hex_2_NeuAc_2_ structure shown. “P” inside a circle corresponds to the peptide backbone of 320-VQPTESIVR-328. The insets correspond to zoom scan from the regions of (**i**) m/z 801, (**ii**) m/z 1004, (**iii**) m/z 474 and (**iv**) m/z 677.

**Fig. 4:**
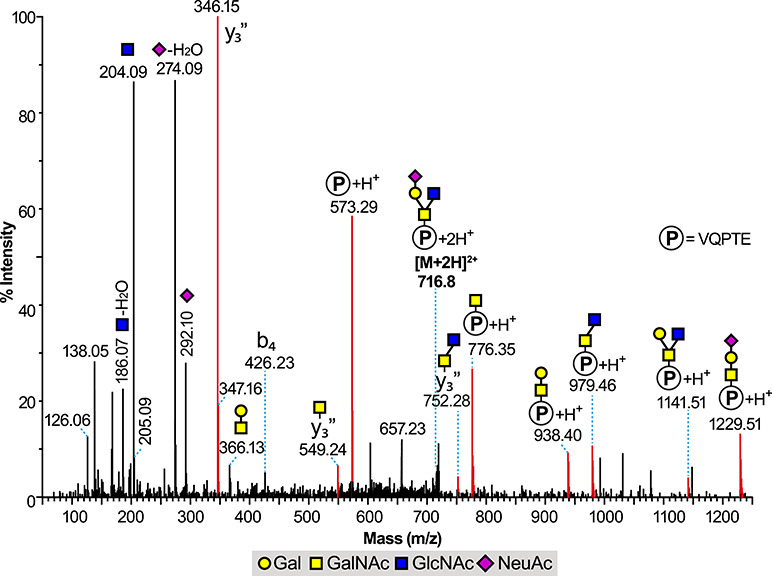
Glycoproteomics analysis of the 320-VQPTE-324 glycopeptide derived from the RBD of the S1 spike SARS-CoV-2 glycoprotein. LC ES-MS/MS data (CAD MS/MS mode) of a doubly charged ion species (716.8^2+^), showing clear evidence of T323 substitution with a mono-sialyl version (NeuAcHexHexNAc_2_) of the assigned Core 2 structure seen in Figure 3 and described in the text. Proline cleavage y” ions at m/z 346, 549 (y_3_-HexNAc) and 752 (y_3_-HexNAc-HexNAc) support the interpretation made. The unlabeled higher mass signals were devoid of ^13^C isotope clusters and not assigned as of glycopeptide origin.

Significant reporter ion activity (m/z 204, 366, 274 and 292) was also observed in the MS^e^ trace of the LC–MS total ion chromatogram between the two regions identified as N-linked sites above. A variety of O-linked glycopeptides were found in the region between 18 and 24 min and the MS^e^ data allowed the identification of several glycoforms of the same 1028.57 (M + H)^+^ peptide which maps onto the N-terminus of the RBD domain as 320-VQPTESIVR-328. The level of O-glycosylation occupancy of this glycopeptide was high, based on the TIC responses of the glycosylated species compared to the nonglycosylated peptide, although it should be noted that without access to validated synthetic materials the ionization efficiencies of respective peptide and glycopeptide counterparts cannot be assumed to be equivalent. Major species in the TIC and XIC traces were identified via 988.4^2+^ eluting at 23.08 min, corresponding to a disialyl Core 1 structure, and via 1171.02^2+^ eluting at 22.45 min corresponding to a NeuAc_2_Hex_2_HexNAc_2_ substitution. Two notable features of the identified peptide are firstly that the Glutamic acid has either not cleaved or only partially cleaved during the Glu-C enzyme treatment, possibly due to steric hindrance of the O-Glycan, and secondly that there are two potential O-linked sites for this peptide at Thr 323 and Ser 325. The MS^e^ spectrum of the 1171.02^2+^ glycopeptide is presented in Figure 3, which shows significant fragment ions above the 1028.57 peptide at m/z 1231.65, 1393.70, 1434.72, 1596.76, 1684.76,1758.78, 1887.79, 2049.78 and 2340.67, indicating a Core 2 substitution with two NeuAcHex antennae, as depicted in Figure 3. Confirmatory data are seen in an intense y” ion observed for the Proline cleavage at m/z 801 (y_7_”), being a fragmentation series continuing through m/z 1004 (+HexNAc) and beyond. A y_4_” ion is observed at m/z 474 for SIVR but there is, however, no evidence of a HexNAc addition to 474 (at m/z 677) above the background signals, indicating that the glycosylation of this peptide is exclusively on the Threonine residue and not the Serine (Figure 3, insets). The facile loss of O-linked glycans from Serine and Threonine within the ion source of a mass spectrometer is well known, however, due to the relative weakness of the O-glycosidic bond (Dell and Morris 2001) coupled with the availability of a beta hydrogen atom to facilitate elimination, and the need to confirm the above finding prompted us to screen the LCMS chromatogram for any species resulting from the expected cleavage at Glu 324, albeit partial (as shown by the dominance of the 1171.02^2+^ ion), to give two potential glycopeptides 320-VQPTE-324 and 325-SIVR-328. Figure 4 shows the CAD MS/MS spectrum of a doubly charged ion at m/z 716.8, corresponding to a molecular ion [M + H]^+^ of 1432.6 eluting at 10.8 min, and the fragmentation interpretation clearly shows a strong molecular ion for the peptide portion of the molecule at m/z 573.29 together with the addition of one and two HexNAcs at m/z 776.35 and 979.46 and HexHexNAc_2_ at m/z 1141.51. A further NeuAcHexHexNAc fragment series (m/z 938.4, 1229.51) allows the overall interpretation of a Core 2 structure with a NeuAcHex antenna on peptide VQPTE for this particular 10.8 min species in Figure 4. Proline y” ions beginning at m/z 346.15 support the assignment. Evidence for the full NeuAc_2_Hex_2_HexNAc_2_ Core 2 structure on peptide 320-VQPTE-324 was found in data within the elution region of the N343 glycopeptide glycoforms, which has clearly led to signal suppression. Nevertheless, reasonable confirmatory data are found, as seen in Supplementary Figure S2 where the key fragment ions arising from the VQPTE glycopeptide are shown, culminating in a molecular ion at m/z 1885.67. Searches for glycosylated SIVR peptides, including data from Glu-C redigested material, proved negative, with the free SIVR peptide itself found at 6.5 min, although simple substitutions such as HexNAc cannot be ruled out because of facile cone-voltage loss from some small glycopeptides.

These results are in contrast to those of Shajahan et al. (2020) who report a Sialyl Core 1 structure on each of Thr 323 and Ser 325 for this O-linked HexNAc_2_Hex_2_NeuAc_2_ glycopeptide (Shajahan et al. 2020). While the fragmentation data in Figure 3 cannot discount that alternative, the site-specific data in Figure 4 and Supplementary Figure S2 support our interpretation of an extended Core 2 structure exclusively on the Threonine 323 residue of the recombinant SARS-Cov-2 S1 RBD reported here. It should be noted of course that the glycosylation may be different in the differing constructs used in these respective studies. Alternatively, we note that if conditions were favorable in the desolvated gas phase for an intramolecular attack of the Ser 325 Hydroxyl on C-1 of the second HexNAc (GlcNAc) of our Core 2 structure (Figure 3), then the result could be two tri-saccharides (NeuAcHexHexNAc) one on each of Thr 323 and Ser 325. This would, however, be an artifact of the experimental method, possibly created by long ion residence trap times or other parameters.

## Discussion

Defining the glycosylation of the SARS-CoV-2 spike glycoprotein is of great importance to better understand virus infectivity, to develop future vaccines and to improve diagnostics. Of particular interest is the RBD since glycosylation sites at N331 and N343, which are found on the RBD of the S1 subunit, together with any O-linked structures, could potentially affect the binding to the ACE2 receptor. We demonstrate that the N-linked sites are glycosylated with complex-type N-glycans with unusual fucosylated LacdiNAc structures, and Thr 323 carrying principally disialyl Core 1 and NeuAc_2_Hex_2_HexNAc_2_ Core 2 O-linked structures. It is well established that HEK293 cells have the biosynthetic potential to generate LacdiNAc containing N-glycans (Do et al. 1997; Dewal et al. 2015). The sites of glycosylation determined in this paper are shown in their relative positions in the RBD amino acid sequence in Supplementary Figure S3.

A great deal of progress in the characterization of SARS-CoV-2 spike glycoprotein has been made in a relatively short time frame. Shajahan et al. (2020) have recently carried out glycoproteomic characterization of the S1 and S2 subunit of the spike glycoprotein individually expressed in HEK293 cells. Their structural analysis indicated that the N-glycans occupying the N331 and N343 glycosylation sites consisted of high-mannose (Man_5-8_GlcNAc_2_) and low molecular weight complex structures. In the work presented here, even though the RBD of the S1 subunit is also expressed in HEK293 cells, we have detected a different N-glycan repertoire occupying both the N331 and N343 glycosylation sites, with exclusively complex N-glycans, lacking high-mannose structures. While some of our O-linked assignments are the same (e.g. diSialyl Core 1 on T323) others differ and we find no clear evidence of S325 glycosylation. In other recent work (Watanabe et al. 2020), glycoproteomic analysis was performed on soluble stabilized trimeric spike glycoprotein (encoding residues 1–1208) expressed in HEK FreeStyle293F cells with substitutions on the furin cleavage site and a C-terminal T4 fibritin trimerisation motif. Interestingly, their proposed N-glycan structural analysis for the N331 and N343 glycosylation sites was more similar to that shown here, in that both glycosylation sites were occupied mainly by complex N-glycans. Some high-mannose N-glycans were also detected, though of a minor abundance. More importantly, LacdiNAc containing N-glycan structures were not reported even though N-glycan compositions matching to these types of glycans were found. This emphasizes the need for detailed N-glycan analysis, which is best achieved by an integrated glycomic and glycoproteomic approach. Although Watanabe et al. (2020) showed some evidence for trace levels of O-glycosylation on a glycopeptide containing both T323/S325, they were not able to achieve site-specific characterization.

It is important to point out, especially to the broader research community, the caveats associated with both our data and the other published S glycoprotein analyses. The actual nature of the analyzed recombinant S glycoprotein varies between the three studies. The RBD region which we analyzed is, as its name suggests, believed to be the most relevant to ACE2 binding, but is a small section of the native viral glycoprotein, whereas the stabilized trimeric spike glycoprotein is most similar to the native viral glycoprotein. However, all are expressed as soluble products and therefore may not have undergone the same biosynthetic maturation as native viral spike glycoprotein. This is likely to impact on both glycan site occupancy and glycan structures. All the recombinant constructs are expressed in cultured HEK cells, but individual cell clones and culture conditions can impact recombinant glycoprotein glycosylation (Zhang et al. 2016). While these are human derived cells, again it is likely that native viral spike glycoprotein, produced by in vivo infection of for example ciliated nasal epithelial cells, will again demonstrate variability in both glycan site occupancy and glycan structures. With the guidance obtained from current studies including the work presented here, future research efforts need to focus on both the production and characterization of S glycoprotein from human cells more similar to those that are actually infected in vivo and to purify and characterize virus-derived S glycoprotein. It is sobering to consider that there was a gap of 16 years between the first glycoproteomic characterization of human-immunodeficiency-virus (HIV) recombinant envelope glycoprotein gp120 (Zhu et al. 2000) and actual virion-derived HIV-1 gp120 (Panico et al. 2016). Given the scale of the current crisis, this cannot be allowed to happen for SARS-CoV-2 spike glycoprotein.

During the review period of our manuscript, work by Zhao et al. (2020), which also details the glycomic/glycoproteomic characterization of trimer-stabilized, soluble SARS-CoV-2 Spike glycoprotein produced in HEK cells, has been published. Their data are consistent with ours in that they confirm the presence of LacdiNAc-containing N-glycan structures and that T323 is the main site of O-glycosylation (Zhao et al., 2020).

## Supplementary Material

Glycan_characterisation_of_SARS-CoV-2_RBD_sites_Supplementary_cwaa085
