# Supplementary material for: Site-specific characterization of SARS-CoV-2 spike glycoprotein receptor-binding domain
Source: Glycobiology. 2020 Sep 3;31(3):181–7. doi: 10.1093/glycob/cwaa085 (PMC7499654; doi:10.1093/glycob/cwaa085)
Supplement: Glycan_characterisation_of_SARS-CoV-2_RBD_sites_Supplementary_cwaa085 [file glycan_characterisation_of_sars-cov-2_rbd_sites_supplementary_cwaa085.pdf]

Site-specific characterisation of SARS-CoV-2 spike glycoprotein receptor binding domain

Keywords: Glycoproteomics / Mass Spectrometry / SARS-CoV-2 / Spike Glycoprotein

Running title: Glycan characterisation of SARS-CoV-2 RBD sites

Aristotelis Antonopoulos[a], Steven Broome[b], Victor Sharov[b], Christopher Ziegenfuss[b] Richard L. Easton[c], Maria Panico[a, b, c], Anne Dell[a], Howard R. Morris[a, b, c], & Stuart M. Haslam[a, 2]

Supplementary data

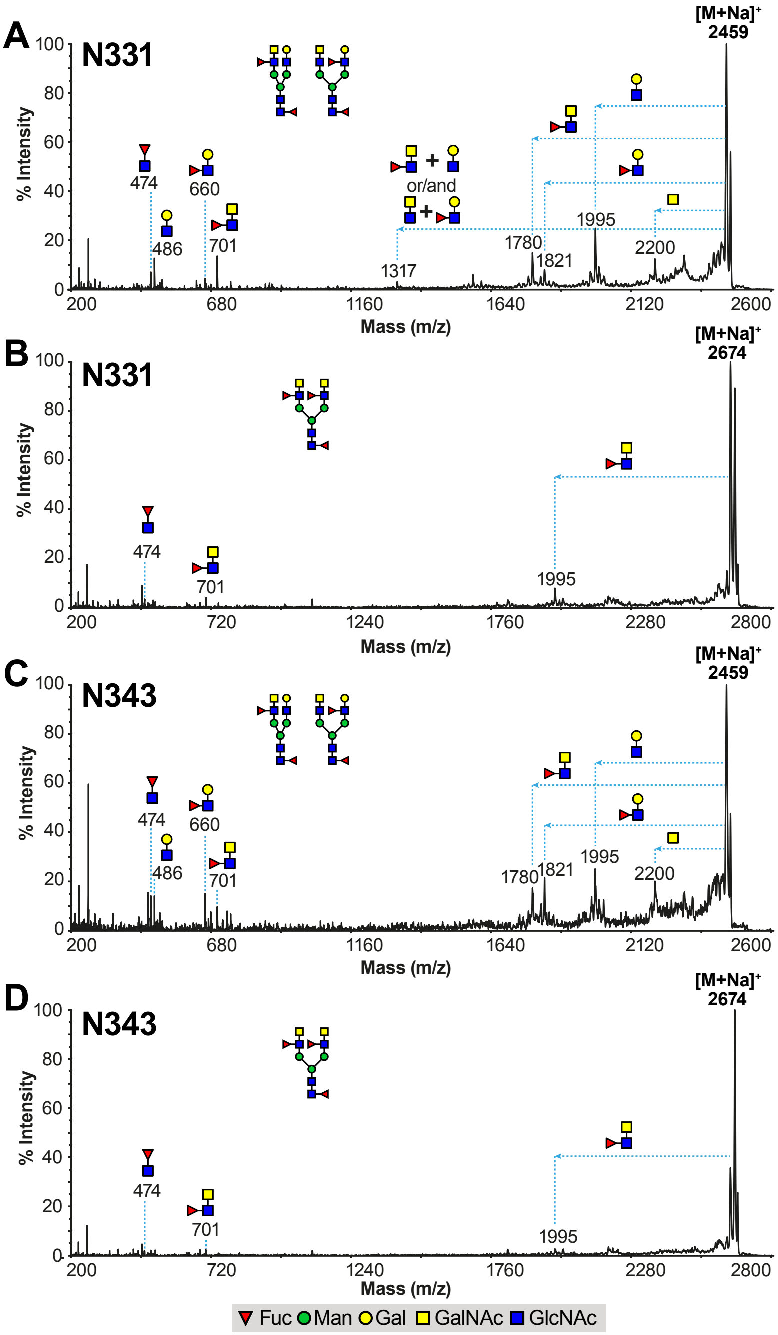
A
N331
[M+Na]+
2459
% Intensity
Mass (m/z)
474
486
660
701
or/and
1317
1780
1821
1995
2200
B
N331
[M+Na]+
2674
474
701
1995
C
N343
[M+Na]+
2459
474
486
660
701
1780
1821
1995
2200
D
N343
[M+Na]+
2674
474
701
1995
Fuc
Man
Gal
GalNAc
GlcNAc

Supplementary Figure S1. MALDI-TOF/TOF MS/MS spectra of selected permethylated N-glycans isolated from the purified glycopeptides N331 and N343 from the recombinant SARS-CoV-2 S1 RBD expressed on HEK293 cells. Selected molecular ions $[M+Na]^+$ are from N331 glycosylation site (**A**) m/z 2459 and (**B**) m/z 2674 (selected from **Figure 1A**), and from the N343 glycosylation site (**C**) m/z 2459 and (**D**) m/z 2674 (selected from **Figure 1B**). All ions are $[M+Na]^+$. Horizontal dashed lines with arrows correspond to indicated losses from the molecular ion $[M+Na]^+$ of the designated N-glycan sequences in insets.

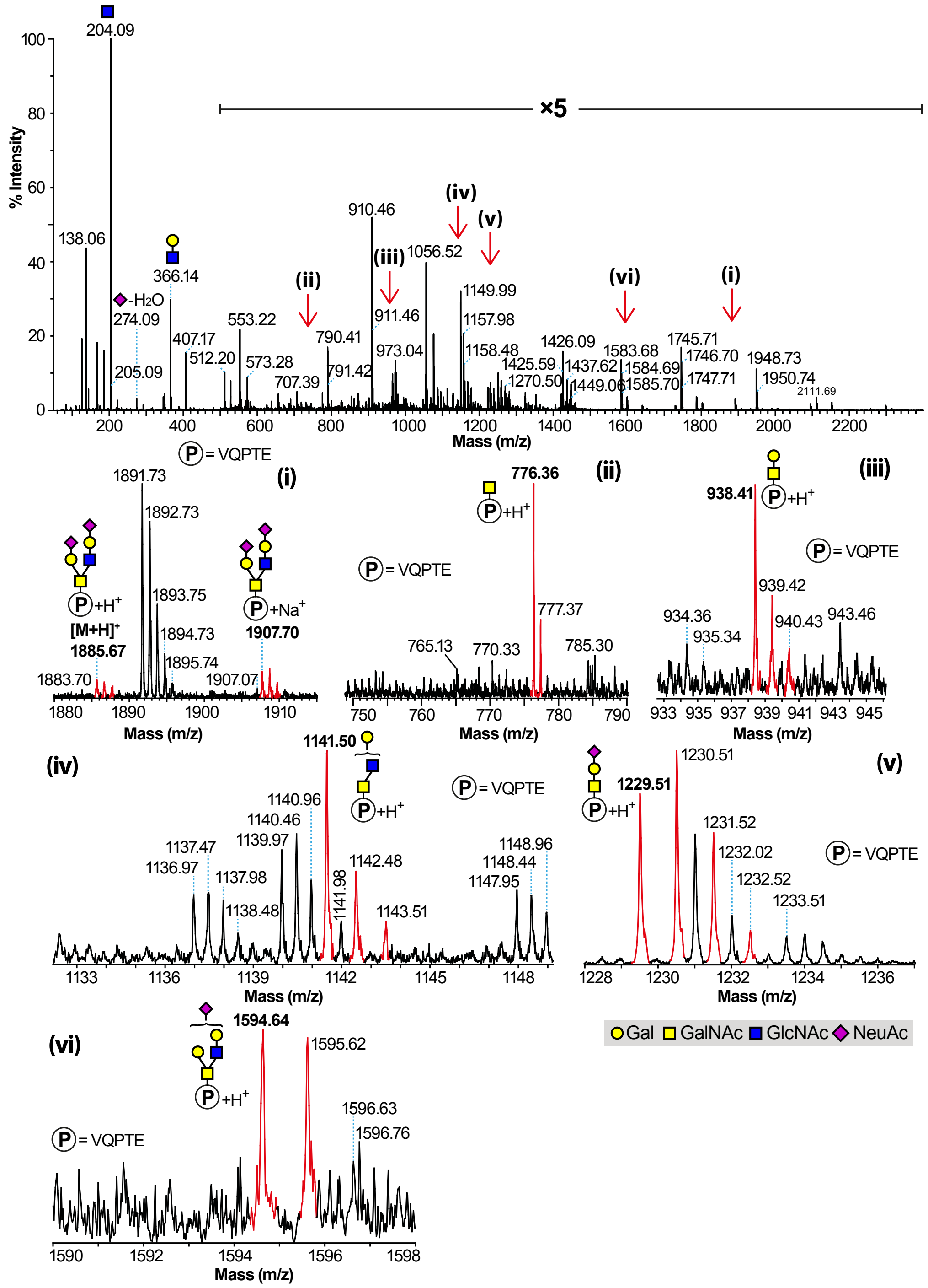
% Intensity
Mass (m/z)
×5
204.09
138.06
366.14
-H2O
274.09
910.46
1056.52
1149.99
(i)
(ii)
(iii)
(iv)
(v)
(vi)
P = VQPTE
[M+H]+
1885.67
1891.73
1907.70
776.36
938.41
1141.50
1229.51
1230.51
1594.64
1595.62
Gal GalNAc GlcNAc NeuAc

Supplementary Figure S2. Glycoproteomics analysis of the 320-VQPTE-324 (Tryptic/Glu-C) glycopeptide containing the glycosylation site T323 derived from the RBD of the S1 spike SARS-CoV-2 glycoprotein found centred at 12.1mins in the LC elution profile. LC ES-MS/MS (MS^e mode) data showing the co-elution of this O-linked glycopeptide with the N-linked glycopeptide described in the text and **Figure 2**. Differentiating the fragmentation series allows identification of the O-linked signals shown in the ascending mass regions, culminating in a molecular ion at m/z 1885.7, providing evidence for the Core 2 $NeuAc_2Hex_2HexNAc_2$ glycoform shown, substituted on Threonine 323. Red arrows in top panel correspond to areas where insets are shown in the lower panel. (**i**) Peaks in red correspond to protonated and sodiated molecular ion (m/z 1885.67 and 1907.70 respectively). **(ii-vi**) Peaks in red correspond to glycopeptide fragment ions deriving from the $[M+2H]^+$ molecular ion. “P” inside a circle corresponds to the peptide backbone of 320-VQPTESIVR-328.

```
                                   320                                     340
                                    |                                       |
                                   RV  QPTESIVRFP NITNLCPFGE VFNATRFASV
                360                                    380                                     400
                 |                                      |                                       |
YAWNRKRISN CVADYSVLYN SASFSTFKCY GVSPTKLNDL CFTNVYADSF
                                   420                                     440
                                    |                                       |
VIRGDEVRQI APGQTGKIAD YNYKLPDDFT GCVIAWNSNN LDSKVGGNYN
                460                                    480                                     500
                 |                                      |                                       |
YLYRLFRKSN LKPFERDIST EIYQAGSTPC NGVEGFNCYF PLQSYGFQPT
                                   520                                     540
                                    |                                       |
NGVGYQPYRV VVLSFELLHA PATVCGPKKS TNLVKNKCVN F
```

Supplementary Figure S3. Amino acid sequence of the Spike protein RBD residues Arg319 to Phe541. The observed sites of N-glycosylation are shown in bold red and the observed site of O-glycosylation is shown in bold green.

Supplementary Table SI. N-glycan compositions observed for the tryptic N-glycopeptide 341-VFNATR-346 (N343) from the recombinant SARS-CoV-2 S1 RBD expressed on HEK293 cells. This table shows the main signals observed consistent with glycopeptides and not an exhaustive list. Some signals may be from cone voltage induced fragmentation in the source of the mass spectrometer.

**Glycopeptide mass observed by LC-ESI/MS $[M+2H]^{2+}$**	**Glycopeptide mass calculated**	**Permethylated glycan mass observed by MALDI-TOF MS $[M+H]^{+}$**	**Composition**
873.36	873.38		$Hex_3HexNAc_2dHex_1$
974.92	974.92		$Hex_3HexNAc_3dHex_1$
1047.92	1047.95		$Hex_3HexNAc_3dHex_2$
1055.94	1055.94		$Hex_4HexNAc_3dHex_1$
1076.46	1076.46	1835.9	$Hex_3HexNAc_4dHex_1$
1128.98	1128.98		$Hex_4HexNAc_3dHex_2$
1149.49	1149.49		$Hex_3HexNAc_4dHex_2$
1157.48	1157.49	2040.0	$Hex_4HexNAc_4dHex_1$
1178.01	1178.00	2081.0	$Hex_3HexNAc_5dHex_1$
1201.49	1201.50		$Hex_4HexNAc_3dHex_1NeuAc_1$
1222.5	1222.52		$Hex_3HexNAc_4dHex_3$
1230.51	1230.51	2214.1	$Hex_4HexNAc_4dHex_2$
1324.0	1324.06		$Hex_3HexNAc_5dHex_3$
1238.52	1238.52	2244.2	$Hex_5HexNAc_4dHex_1$
1259.02	1259.03	2285.1	$Hex_4HexNAc_5dHex_1$
1303.01	1303.04	2401.2	$Hex_4HexNAc_4dHex_1NeuAc_1$
1311.55	1311.54	2418.2	$Hex_5HexNAc_4dHex_2$
1332.05	1332.06	2459.2	$Hex_4HexNAc_5dHex_2$
1384.06	1384.06	2605.3	$Hex_5HexNAc_4dHex_1NeuAc_1$
1404.57	1404.58	2646.3	$Hex_4HexNAc_5dHex_1NeuAc_1$
1405.07	1405.09	2633.3	$Hex_4HexNAc_5dHex_3$
1425.59	1425.60	2674.3	$Hex_3HexNAc_6dHex_3$

1457.08	1457.09	2779.4	$Hex_5HexNAc_4dHex_2NeuAc_1$
1477.59	1477.61	2820.4	$Hex_4HexNAc_5dHex_2NeuAc_1$
1485.58	1485.60	2850.4	$Hex_5HexNAc_5dHex_1NeuAc_1$
1529.60	1529.61	2966.4	$Hex_5HexNAc_4dHex_1NeuAc_2$
1566.61	1566.63	3054.5	$Hex_6HexNAc_5dHex_1NeuAc_1$
1631.12	1631.15	3211.5 (trace)	$Hex_5HexNAc_5dHex_1NeuAc_2$
1639.62	1639.66	3589.8 (trace, peak top mass)	$Hex_6HexNAc_5dHex_2NeuAc_1$

Supplementary Table SII. N-glycan compositions observed for the tryptic N-glycopeptide 329-FPNITNLCPFGE-340 (N331) from the recombinant SARS-CoV-2 S1 RBD expressed on HEK293 cells. This table does not give an exhaustive list of signals observed consistent with glycopeptides. Some glycopeptide signals observed are likely to be from cone voltage induced fragmentation in the source of the mass spectrometer.

**Glycopeptide mass observed by LC-ESI/MS $[M+3H]^{3+}$**	**Glycopeptide mass calculated**	**Permethylated glycan mass observed by MALDI-TOF MS $[M+H]^{+}$**	**Composition**
952.06	952.06	1835.9	$Hex_3HexNAc_4dHex_1$
1006.08	1006.08	2040.1	$Hex_4HexNAc_4dHex_1$
1019.76	1019.78	2081.1	$Hex_3HexNAc_5dHex_1$
1054.77	1054.77	2214.1	$Hex_4HexNAc_4dHex_2$
1060.09	1060.10	2244.2	$Hex_5HexNAc_4dHex_1$
1068.43	1068.44	2255.2	$Hex_3HexNAc_5dHex_2$
1073.76	1073.77	2285.2	$Hex_4HexNAc_5dHex_1$
1108.78	1108.78	2418.3	$Hex_5HexNAc_4dHex_2$
1122.46	1122.46	2459.3	$Hex_4HexNAc_5dHex_2$
1136.13	1136.14	2500.3	$Hex_3HexNAc_6dHex_2$
1157.13	1157.13	2605.4	$Hex_5HexNAc_4dHex_1NeuAc_1$
1170.80	1170.80	2646.4	$Hex_4HexNAc_5dHex_1NeuAc_1$
1171.15	1171.15	2633.4	$Hex_4HexNAc_5dHex_3$
1184.84	1184.82	2674.4	$Hex_3HexNAc_6dHex_3$
1205.81	1205.82	2779.4	$Hex_5HexNAc_4dHex_2NeuAc_1$
1219.48	1219.49	2820.5	$Hex_4HexNAc_5dHex_2NeuAc_1$
1254.16	1254.17	2966.5	$Hex_5HexNAc_4dHex_1NeuAc_2$
1278.84	1278.84	3054.6	$Hex_6HexNAc_5dHex_1NeuAc_1$

Supplementary Table SIII. O-glycan compositions observed for the tryptic O-glycopeptide 320-VQPTESIVR-328 from the recombinant SARS-CoV-2 S1 RBD expressed in HEK293 cells. Some signals may be from cone voltage induced fragmentation in the source of the mass spectrometer.

**Glycopeptide mass observed by LC-ESI/MS $[M+2H]^{2+}$**	**Glycopeptide mass calculated**	**Composition**
616.33	616.33	HexNAc
798.89	798.90	$HexHexNAc_2$
879.92	879.92	$Hex_2HexNAc_2$
944.44	944.44	$HexHexNAc_2NeuAc$
1025.47	1025.47	$Hex_2HexNAc_2NeuAc$
1171.02	1171.02	$Hex_2HexNAc_2NeuAc_2$
